# Quantitative automated microscopy (QuAM) elucidates growth factor specific signalling in pain sensitization

**DOI:** 10.1186/1744-8069-6-98

**Published:** 2010-12-27

**Authors:** Christine Andres, Sonja Meyer, Olayinka A Dina, Jon D Levine, Tim Hucho

**Affiliations:** 1 Department for Molecular Human Genetics, Max Planck Institute for Molecular Genetics, Ihnestrasse 73, 14195 Berlin, Germany; 2 Institute of Chemistry and Biochemistry, Freie Universität Berlin, Berlin, Germany; 3 Max Planck Institute for Dynamics of Complex Technical Systems and Magdeburg Centre for Systems Biology (MaCS), Otto von Guericke University, Magdeburg, Germany; 4 Division of Neuroscience, Departments of Medicine and Oral & Maxillofacial Surgery, University of California, San Francisco, CA, USA

## Abstract

**Background:**

Dorsal root ganglia (DRG)-neurons are commonly characterized immunocytochemically. Cells are mostly grouped by the experimenter's eye as "marker-positive" and "marker-negative" according to their immunofluorescence intensity. Classification criteria remain largely undefined. Overcoming this shortfall, we established a quantitative automated microscopy (QuAM) for a defined and multiparametric analysis of adherent heterogeneous primary neurons on a single cell base.

The growth factors NGF, GDNF and EGF activate the MAP-kinase Erk1/2 via receptor tyrosine kinase signalling. NGF and GDNF are established factors in regeneration and sensitization of nociceptive neurons. If also the tissue regenerating growth factor, EGF, influences nociceptors is so far unknown. We asked, if EGF can act on nociceptors, and if QuAM can elucidate differences between NGF, GDNF and EGF induced Erk1/2 activation kinetics. Finally, we evaluated, if the investigation of one signalling component allows prediction of the behavioral response to a reagent not tested on nociceptors such as EGF.

**Results:**

We established a software-based neuron identification, described quantitatively DRG-neuron heterogeneity and correlated measured sample sizes and corresponding assay sensitivity. Analysing more than 70,000 individual neurons we defined neuronal subgroups based on differential Erk1/2 activation status in sensory neurons. Baseline activity levels varied strongly already in untreated neurons. NGF and GDNF subgroup responsiveness correlated with their subgroup specificity on IB4(+)- and IB4(-)-neurons, respectively. We confirmed expression of EGF-receptors in all sensory neurons. EGF treatment induced STAT3 translocation into the nucleus. Nevertheless, we could not detect any EGF induced Erk1/2 phosphorylation. Accordingly, intradermal injection of EGF resulted in a fundamentally different outcome than NGF/GDNF. EGF did not induce mechanical hyperalgesia, but blocked PGE_2_-induced sensitization.

**Conclusions:**

QuAM is a suitable if not necessary tool to analyze activation of endogenous signalling in heterogeneous cultures. NGF, GDNF and EGF stimulation of DRG-neurons shows differential Erk1/2 activation responses and a corresponding differential behavioral phenotype. Thus, in addition to expression-markers also signalling-activity can be taken for functional subgroup differentiation and as predictor of behavioral outcome. The anti-nociceptive function of EGF is an intriguing result in the context of tissue damage but also for understanding pain resulting from EGF-receptor block during cancer therapy.

## Background

A common denominator of DRG-neurons is their extreme heterogeneity. They differ in respect to parameters such as morphology, protein expression and functionality [1]. To what extent functional differences can be correlated to expression differences of e.g. ion channels is a matter of intense research. Mostly the expression of a "marker" is detected via immunofluorescence microscopy [2]. The grouping into "marker-positive" and "marker-negative" cells is commonly performed qualitatively by eye by a trained experimenter. That a differentiation into a "positive" and "negative" population can be accomplished by eye is mostly only assumed but not experimentally addressed. Even further, evaluation through an experimenter's eye does not allow a definition of exact quantitative parameters such as which staining intensity qualifies as "positive". Thereby comparability between different labs, experimenters and/or experimental days cannot be analyzed and has to be questioned. The inherent inaccuracy of the "by eye" evaluation of intensities might underlie the wide variety of population sizes reported in the literature. For example the population size of IB4(+)-neurons ranges from 40 - 70% [3-5] and population size for TrkA(+)-neurons ranges from 35 - 70% [3,6]). In addition, more complex evaluations such as the gradual increase in activation of signalling components are near to impossible. This is in times of highly sophisticated means of quantification increasingly unsatisfying and problematic. Thus, we set out to establish a technique to detect and quantify immunofluorescence signals of adherent primary sensory neurons on a single cell base.

Functionality of sensory neurons is mostly addressed by investigation of ion channel properties and differential ion channel expression. But the sensitivity of ion channels can be modulated to a great extend by post translational modification such as phosphorylation in the course of intracellular signalling cascade events [1,7-9]. Therefore activation of a receptor not necessarily results in the activation of its downstream signalling. Thus the expression of a receptor cannot be taken as synonymous with functional changes in these neurons. To what extent the activation properties of intracellular signalling cascades themselves can be taken to differentiate functional different neuronal subgroups has not been addressed so far. Thus we tested if we can detect gradual increase in signalling component activation in subgroups of sensory neurons and if investigation of such cellular signalling kinetics allows to predict if a substance has a sensitizing effect in behavioral experiments.

Growth factors play a dual role in sensory neurons. In tissue challenged with potentially damaging stimuli they mediate neuronal tissue defense and alarm of the organism. NGF is a potent endogenous stimulator of neuronal survival and nerve fiber growth [10-12] and is essential for reinnervation of the skin after injury to cutaneous nerves [13,14]. But NGF also initiates and maintains hypersensitivity [15]. Also the growth factor, GDNF, shows this dual effect. On one hand GDNF is an essential growth factor for the survival and functional maintenance of a subgroup of nociceptors during development and after insult [16,17]. On the other hand GDNF has been reported to contribute to inflammatory hyperalgesia in an adjuvant-induced pain model [18] and also acutely if injected into the skin [19].

On the cellular level, growth factors bind and activate receptor tyrosine kinase receptors, resulting among others in the activation of the MAPK Erk1/2 [20,21]. Accordingly, GDNF and NGF-induced mechanical pain sensitization has been identified to depend on the activation of Erk1/2 [19,22].

Beyond the known effects of the growth factors NGF and GDNF on nociceptors, there is another family of growth factors central to tissue protection and wound healing, the EGF-family [23]. They are secreted after tissue injury by platelets, macrophages and fibroblasts to initiate proliferation and regeneration of the epithelium [24,25]. Wound healing can be improved by EGF administration [26,27]. Like NGF, EGF exerts its cellular action via a member of the receptor tyrosine kinase family, the EGFR. The receptors of NGF and EGF share many structural features as well as activation associated intracellular signalling cascades. One signalling component reported in non-nociceptive cells to be central to EGF-as well as NGF/GDNF signalling is again Erk1/2 [28].

While described as a survival and growth factor, it is not known, if EGF has the potential to act on nociceptive neurons. Accordingly, it is also unknown if EGF like NGF and GDNF sensitizes them. As all three growth factors act by activation of Erk1/2 this appears likely. But of special interest is a study in PC12 cells indicating that the outcome of NGF and EGF treatment can be very different. While stimulation with NGF leads to neurite outgrowth and differentiation, stimulation with EGF leads to the diametral opposing cellular response, namely cell proliferation [29]. Interestingly, both actions were described to be mediated by activation of Erk1/2. How the very same signalling component can result in two opposing phenotypes has been enigmatic for long. Only recently the opposing phenotypic outcome of NGF and EGF signalling was correlated to a differential stimulation kinetic of Erk1/2 phosphorylation induced by NGF and EGF. While NGF results in a slower but more pronounced and long-lasting activation, EGF activates Erk1/2 earlier but only transiently [30]. Thus a cellular phenotypic outcome can only in part be derived from the activation of mediating signalling components as such. Equally important aspects are the kinetic parameters like the amplitude, the pace of activation-onset as well as the duration of activation. Due to the lack of appropiate techniques it has not been analyzed in nociceptive neurons if there are differences in the kinetics of Erk1/2 in response to NGF versus EGF.

Therefore‚ we investigated if nociceptive neurons express the EGF-receptor and if EGF results in activation of Erk1/2 similar to the growth factors NGF and GDNF. We thereby tested the concept if the comparison of cellular activation responses can be used for predicting if EGF sensitizes nociceptors in behavioral experiments. For investigating signalling kinetics of endogenous signalling components of nociceptive neurons on single neuron base we introduced a quantitative automated microscopy (QuAM).

## Methods

### Chemicals

BSA, L-glutamine, poly L-ornithine hydrochloride, DMSO, paraformaldehyde, Triton X-100 and glutamate were purchased from Sigma (Taufkirchen, Germany), collagenase P from Roche (Mannheim, Germany), trypsin from Worthington Biochemical Corporation (Freehold, NJ, USA), Neurobasal A (without phenol red), B27 supplement, laminin, minimum essential medium with glutamax were purchased from Invitrogen (Germany, UK), DMEM, trypsin and EDTA from Clonetics (Cambrex, US) and normal donkey serum from Dianova (Hamburg, Germany).

### Drugs

PMA, EGF, PGE_2 _and epinephrine were purchased from Sigma (Taufkirchen, Germany or St. Louis, MO). mNGF was purchased from Alomone (Jerusalem, Israel), GDNF was purchased from PeproTech (Hamburg, Germany).

### Antibodies

Anti-PGP 9.5 was purchased from MorphoSys AG (Martinsried/Planegg, Germany).

Anti-phospho-Erk (Thr-202/Tyr-204) and anti-phospho-STAT3 (Tyr705) were purchased from New England Biolabs (Frankfurt am Main, Germany, f.c. 1:200 and 1:100 respectively). Anti-Erk1/2 (pan) was purchased from BD Bioscience (Heidelberg, Germany, f.c. 1:500) Anti-EGFR and an EGFR-C-terminal peptide were purchased from Abcam (Cambridge, UK, f.c. Anti-EGFR 1:500).

Alexa-594-labeled chicken anti-rabbit IgG and Alexa-633 goat anti-mouse IgG were purchased from Molecular Probes/Invitrogen (Karlsruhe, Germany; f.c. 1:1000 for immunocytochemistry, 1:500 for immunohistochemistry). FITC-coupled anti-mouse IgG was purchased from Dianova (Hamburg, Germany; f.c. 1:500 for immunocytochemistry and 1:200 for immunohistochemistry). FITC labeled IB4 (f.c. = 1 ng/μl) and unlabeled IB4 (f.c. 100 ng/μl) were purchased from Sigma (Taufkirchen, Germany).

### DRG-cultures

Cultures of dissociated DRG were prepared from male Sprague Dawley rats as described previously [31]. The rats were killed by CO_2 _intoxication and L1-L6 DRGs were removed, desheathed, pooled and incubated with collagenase (final concentration (f.c.) 0.125%; 1 h, 37°C). The neurons were dissociated by trypsin digestion (f.c. 0.25%, 1176 u, 8 min, 37°C) and triturated with a fire-polished Pasteur pipette. Axon stumps and dead cells were removed by centrifugation (5 min, 100 g). Viable cells were resuspended in 12 ml of NeurobasalA/B27 medium, plated 0.5 ml/culture onto polyornithine/laminin-precoated glass coverslips (12 mm diameter), and incubated overnight in 24 well plates at 37°C in 5% CO_2_.

### Cell stimulation

After incubation for 15-20 h cells were stimulated with the growth factors NGF, GDNF, EGF or pharmacologically with Phorbol 12-myristate 13-acetate (PMA), respectively. To ensure homogeneous mixture of the stimulants, a volume of 250 μl out of the 500 μl culture medium was removed from the culture well, mixed thoroughly with the stimulant, and added back to the same culture. Negative controls were treated alike but without the addition of any reagent. To reduce mechanical cell stress the stimulus was added very slowly (250 μl in 6 s) using an automatic pipette (Multipette^® ^pro, Eppendorf). After treatment, the cells were washed once with phosphate-buffered saline (PBS) and fixed with paraformaldehyde (4%, 10 min) at room temperature (RT). For staining with the pSTAT3 antibody cells were fixed and permeabilized with methanol (10 min, -20°C)

### Immunocytochemistry

Paraformaldehyde-fixed cells were permeabilized with 0.1% Triton X-100 (10 min, RT), followed by three washes with PBS (5 min, RT). After blockage of nonspecific binding sites (5% bovine serum albumin (BSA) and 10% normal donkey serum in PBS; 1 h, RT), the cultures were probed with primary antibodies against target proteins (antibody concentrations against target proteins as indicated in Methods, Antibodies section) in 1% BSA in PBS (1 h, RT), washed three times (1% BSA in PBS; 5 min, RT), and incubated with secondary antibodies (1 h, RT). After three final washes (PBS; 5 min, RT), the cultures were mounted with Fluoromount-G (Southern Biotech⁄ Biozol) containing DAPI (0.5 μg/ml). Staining with the isolectin IB4 from *Bandeiraea simplicifolia *was performed in a solution containing 0.1 mM Ca^2+^, 0.1 mM; Mg^2+ ^and 0.1 mM Mn^2+ ^in PBS (1 h, RT), followed by three washes (0.1 mM Ca^2+^, 0.1 mM; Mg^2+ ^and 0.1 mM Mn^2+ ^in PBS, 5 min, RT) before mounted onto microscopy slides with DAPI/Fluoromount-G.

### Immunohistochemistry

Prepared L3-L6 DRGs were fixed with paraformaldehyde (4%, 1 h, RT), washed 3× with PBS (20 min, RT), saturated by increasing concentrations of sucrose (10, 20 and 30%, each saturation step overnight, 4°C) and embedded in Tissue tek^® ^(EMS Science Services). Material was frozen on dry ice and stored at -80°C. DRGs were cut in 20 μm sections using a Cryostar Cryostat HM560 and dried on microscope slides (30 min, 37°C). The sections were fixed by paraformaldehyde (4%, overnight, 4°C). Immunostaining was performed as indicated in the "Immunocytochemistry" section.

### QuAM

Cells were evaluated with a Zeiss Axioplan 2 microscope controlled by the software Metacyte (Metasystems). Images of 1280 × 1024 pixels were taken using a 10× objective. The exposure time was defined automatically so that maximal 1000 pixel/100 μm^2 ^reached saturation (i.e. maximal intensity values), but was maximal 0.96s. For automatic neuron recognition the following parameters were defined: size (150 -1500 μm^2^), form (aspect ratio = 2; concavity depth = 0.25), contrast (object threshold 30%). The integrative pixel intensity of each selected neuron was normalised against the respective neuron area and exposure time. The influence of varying exposure times on the resulting intensity value is insignificant (Additional File 1: Figure S1). For cell identification the neuron specific PGP 9.5 immunostaining was used as independent selection marker. Fluorescence intensities derived from phospho-Erk1/2 antibody and/or IB4-signals were quantified on independent color channels.

### Random sampling

Our single cell measurement data are not distributed normally as they neither pass a normal distribution test nor a chi-square test. Thus, statistical comparisons of untreated and treated cultures were performed using a random sampling approach. "Virtual culture wells" were created by randomly picking 250, 1000, 5000, 10000 or 20000 cells out of all measured cells and the average intensity of these virtual wells was computed. Sampling and computing the average was repeated 10,000 times. One and the same cell-value could be picked more than once. The sample mean is, by the Central Limit Theorem, approximately normally distributed, with mean equal to the population mean.

### Confocal microscopy

For evaluation of protein expression confocal images of cells and sections derived from DRG were taken on an inverted Zeiss LSM 510 Meta or a Zeiss LSM 700 with 63× or 40× objectives. Plasma membrane staining was analysed via intensity-histograms with the software ImageJ by measuring the fluorescence intensity profile along a cell crossing line. The intensity of nucleus localized pSTAT3 signal was quantified also with ImageJ.

### Evaluation of pSTAT3 translocation

Cells were evaluated with a Zeiss Axioplan 2 Imaging using a 63× oil-immersion objective. Fifty randomly selected cells per culture were evaluated. Data are plotted as mean percentage of translocating cells per evaluated cultures + SEM based on the number of evaluated cultures. All counting was done by the same observer. All treatments have been repeated three times with DRG-neurons from different rats.

### RT-PCR

For RNA extraction DRGs or brain from male Sprague-Dawley rats were isolated. Total RNA was extracted using the Nucleospin RNA/Protein Kit (Macherey-Nagel, Düren, Germany). DNA was digested for 15 min at 37°C with RQ1 DNAse (Promega, Mannheim, Germany). RNA was precipitated (0.1 volume 3 M sodiumacetate, 2.5 volumes ethanol; 20 min, 20°C and 10 min, 20800 g), washed with 70% ethanol (5 min, 20800 g), dried and resuspended in RNAse-free water. cDNA was created using SuperScript III First Strand Synthesis SuperMix for qRT PCR (Invitrogen, Karlsruhe, Germany). An EGFR-specific fragment was amplified with specific primers (5'- CATCCAGTGCCATCCAGAAT - 3' (forward) and 5'- CTTCCAGACCAGGGTGTTGT - 3' (reverse)) via PCR (2 min at 94°C, 30, 35 or 40 cycles of 30 s at 94°C, 30 s at 53°C, 30 s at 72°C and a final step of 10 min at 72°C). The PCR product was analysed on a 2% agarose gel and imaged with a Herolab EASY 440K gel documentation system.

### Testing of mechanical nociceptive threshold

The nociceptive flexion reflex was quantified using a Randall Selitto paw pressure device (Analgesymeter; Stoelting, Wood Dale, IL), which applies a linearly increasing mechanical force to the dorsum of the rat's hindpaw. The nociceptive mechanical threshold was defined as the force in grams at which the rat withdrew its paw. The protocols for this procedure have been described previously [32,33]. All experiments were performed at the same time of day. In the week preceding the experiments, rats were familiarized with the testing procedure at 5 min intervals for a period of 1 h per day for 3 days. Baseline paw withdrawal threshold was defined as the mean of six readings before test agents were injected. Each paw was treated as an independent measure, and each experiment was performed on a separate group of rats. Each group of rats was treated with only one agonist injected intradermally. The nociceptive threshold was measured 30 min after the administration of the respective reagent. The reagents (see description in the Results section) were injected as described previously [34,35].

### Statistical Analysis

For statistical comparisons of EGFR fluorescence intensities and pSTAT3 nucleus localized fluorescence intensities the Students t-test with Welsh-correction was applied. Statistic analysis of pSTAT3 nucleus translocation as well as comparison of pErk1/2 and Erk1/2 fluorescence intensities between IB4(+)- and IB4(-)-neurons was done with an one-tailed unpaired t-test. For statistical comparison of NGF and GDNF treated vs. untreated cultures the sample mean resulting from the random sampling approach were tested by an one-tailed unpaired t-test. Statistic analysis of behavioral data was done with one-way ANOVAs followed by Dunnett's test for comparisons with one control value. P < 0.05 was considered as statistically significant.

## Results

### Software-based identification of dissociated DRG-neurons

**Figure 1:**
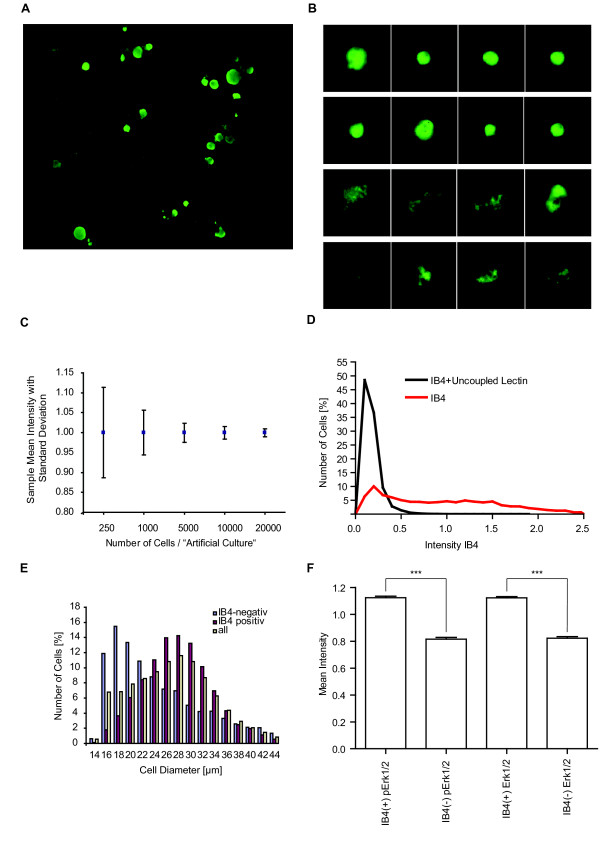
**Automatic DRG-neuron identification and quantification of phosphospecific fluorescence intensities**. A) Example image of a microscope view field of the DRG neuron culture immunofluorescently labelled for the neuronal marker PGP9.5. Neurons show high degree of heterogeneity in respect to size, fluorescence intensity and extend of cell-cell contacts. B) Examples of objects selected as neurons (upper two rows) and rejected objects (lower two rows). Rejected objects include non-cellular debris, glia cells and clustered neurons. C) "Virtual wells" were created by randomly selecting 250, 1000, 5000, 10,000 or 20,000 cells per virtual well out of 49,503 measured cells. The normalized mean well intensity of 10,000 such virtual wells with the respective standard deviation is depicted indicating the sensitivity limit of studies based on the various average cell numbers per well (250 cells per well resulted in a well Mean Intensity of 1.0005 ± 0.1133, 1000 cells in 1.0003 ± 0.0564, 5000 cells in 0.9999 ± 0.0240, 10,000 cells in 0.9997 ± 0.0159, and 20,000 cells result 0.9998 ± 0.0098). D) IB4 lectin derived fluorescence intensities showed a continuous distribution of intensities. Coincubation with uncoupled lectin to block specific binding resulted in an intensity histogram (n = 5002 cells, black line) matching the first peak of the unblocked IB4-intensity histogram (n = 23266 cells, red line) identifying these cells as unspecificly binding and thus as IB4(-)-cells. E) Diameter histogram of 49,503 neurons (yellow bars). 40,706 neurons were labeled for IB4. IB4(+)-neurons (red bars) show a higher average diameter than IB4(-)-neurons (blue bars). F) Comparison of the normalized Mean Intensity of phosphorylated and total Erk1/2 in IB4(+)- and IB4(-)-neurons (n = 1421 IB4(+)-neurons and n = 963 IB4(-)-neurons). IB4(+)-neurons show a higher amount of pErk1/2 but also a higher expression rate of Erk1/2 in comparison to IB4(-)-neurons (p < 0.001).

The heterogeneity of primary DRG-neuron cultures challenges the first step of a quantitative image analysis, the object identification. The DRG-neurons, but not axon stumps or glia cells have to be identified by setting suitable "object identifier" parameters in the image-analysis software. Images of immunofluorescently labeled dissociated DRG-neurons culture were taken by an automated microscope (Figure 1A) and neurons identified by the image analysis software "Metacyte" (Metasystems). Neurons were visualized with an antibody against the neuronal marker PGP 9.5. Object identification parameter optimization was performed and allowed the identification of neurons based on object size (150-1500 μm^2^), roundness (ratio of longest to shortest diameter < 2), smoothness of cell perimeter (concavity depth < 0.25), and relative PGP 9.5 immunofluorescence intensity (> 30% difference to the surrounding) (Figure 1B).

In the culture 16 ± 1% of all cells identified visually as neurons based on their shape and neuronal marker PGP 9.5 expression were in cell clusters. These clustered cells were rejected as cells function very differently if cultured as separate cells or connected to others. Further 3 ± 2% of all neurons were cut by the viewfield borders of the images and were therefore also rejected. Of the remaining 81% of all neurons in culture, 93 ± 2% were detected by the software indicating a very robust and well optimized detection algorithm.

### Single DRG-neuron based studies require large number of analyzed cells per treatment

One signalling component important in nociceptor sensitization is the MAP-kinase Erk1/2 [36]. Activation of Erk1/2 is commonly detected by use of antibodies specific for the Thr-202/Tyr-204 phosphorylation site of Erk1/2. We tracked the basal phosphorylation Erk1/2 signals in untreated cultures with basal levels of activation and investigated 49,502 single cells. The degree of immunofluorescently detected phosphorylation shows an intensity distribution with a standard deviation of ± 180%.

Considering the high standard deviation of intensities we next determined, how many cells have to be measured to describe the distribution sufficiently and what level of sensitivity is reached thereby.

One can estimate, how good the "real" distribution is detected by comparing the distribution of culture wells with small numbers of cells with the distribution of a very large number of measured cells. To do so, out of 49,502 measured unstimulated cells we randomly sampled 10,000 artificial cultures consisting each of 250, 1000, 5000, 10,000 and 20,000 cells per virtual well, respectively (Figure 1C). As expected, sampling more cells per culture resulted in an ever-narrowing degree of deviation of mean-culture-intensities. For 250 cells the standard deviation of the mean is +/- 11%, for 1000 +/- 6%, for 5000 and 10,000 cells +/- 2%, and for 20,000 cells +/- 1%. This indicates, that mean culture intensity changes below 22% (two times standard deviation) intensity change cannot be assumed statistically sound if only 250 cells are investigated. Of note, an intensity increase in only a subgroup of e.g. 1/5 of the cells, requires with 250 measured cells per culture an increase of at least 5 × 22% = 110%, i.e. roughly a doubling of the intensity, within the subgroup to result in an overall mean change of 22%. But investigation of more cells allows the detection of smaller changes even down to some few percent change (standard deviation for 10,000 measured neurons = +/- 2%) indicating high sensitivity of our relative immunofluorescence approach despite the apparent huge heterogeneity of DRG-cultures.

### Subgroups can be defined by classical expression markers

In most published reports of immunofluorescently-labeled marker-protein-based subgroups, the categorization into "marker-positive" and "marker-negativ" is performed by a trained experimenter judging by eye [2,3,37]. This assumes a clear distinction between the positive and the negative cells. This assumption nevertheless has never been tested thoroughly.

One well established marker for such a DRG-neuron subgroup is the isolectin B4 (IB4) identifying preferably non-peptidergic GDNF-dependent nociceptive C fibers. Thus we measured the intensities of single cells labeled with IB4. The intensities showed a continuum of intensities (Figure 1D). Thus the assumption of clearly separated intensity ranges for marker-positive and marker-negative cells is not valid.

Nevertheless, representing the data by an intensity histogram one is tempted to differentiate one population in the lower intensity range (large peak on the left) and a shallow but widespread distribution with much higher intensities (right of the large peak) (Figure 1D). Using an unlabeled lectin we tested, if the low intensity population represents unspecific binding of the lectin and thus is the marker-negative population. The competitive block eliminated all specific IB4-signal and resulted in ablation of the higher intensity part of the histogram. By taking the point of intersection of both intensity histograms as cut-off we identified the two distributions as IB4 positive and IB4 negative neurons (Figure 1D). Analysis of 40,706 cells identified 71 ± 8% to be IB4(+)- and 29 ± 8% to be IB4(-)-neurons.

### The IB4(+)-subgroup shows higher basal Erk1/2 phosphorylation

The potential strength of the quantitative immunofluorescence and single cell based analysis of the heterogenous culture is that one can analyze marker defined subgroups in depth by parameter combination independent of other cells in the very same culture. We performed double staining detecting on one hand the static subgroup marker IB4. On the other hand with antibodies directed against phosphorylated i.e. activated Erk1/2 we investigated the dynamic activation state of Erk1/2. The classified IB4(+)- and IB4(-)-neurons were compared in respect to their size and basal level of Erk1/2 phosphorylation. The majority (63%) of IB4(+)-neurons have diameters in the range between 22-32 μm whereas most IB4(-)-neurons seem to be smaller as 60% have diameters between 14-24 μm (Figure 1E).

We thereby describe not only a size difference between IB4(+)- and IB4(-)-cells but we detected for the first time a difference in the basal level of Erk1/2 phosphorylation. In IB4(+)-cells the mean intensity of pErk1/2 is measured to an intensity value of 1.12 ± 0.01 (Mean ± SEM) versus 0.82 ± 0.001 (Mean ± SEM) in IB4(-)-cells (Figure 1F). Interestingly, the increase in phosphorylated Erk1/2 is matched by an increase in total Erk1/2 expression. Thus, while the relative amount of activated Erk1/2 appears to remain constant, the total amount, i.e. the concentration of activated Erk1/2 per cell increases.

### Functional subsets of neurons can be defined by growth factor induced increases of pErk1/2 intensities on a single cell level

**Figure 2:**
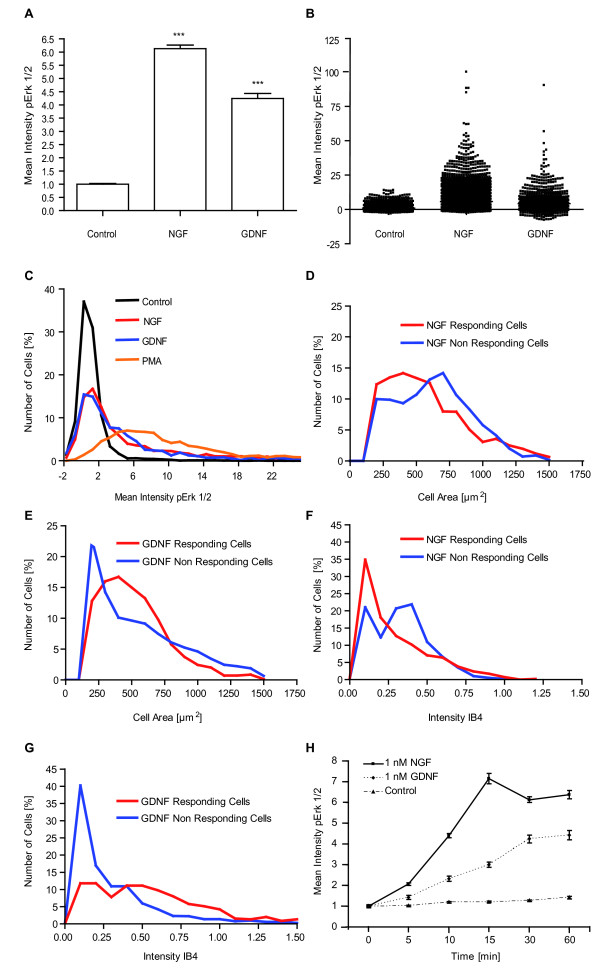
**NGF and GDNF stimulation leads to Erk1/2 phosphorylation in subpopulations**. A) Stimulation with NGF (1 nM, 30 min) and GDNF (1 nM, 30 min) led to significant increase of pErk1/2 intensity levels (n = 5203 cells for no stimulus, n = 3778 cells for NGF treatment, n = 1275 cells for GDNF treatment, error bars are SEM, p < 0.001 for NGF/GDNF vs control). B) 1D scatter plot of the single cell data plotted in A) as bar graphs. Thereby, the huge heterogeneity with up to 100-fold intensity differences is clearly visible. C) Intensity histogram of unstimulated (black line), NGF (red line), GDNF (blue line) and PMA (orange line) stimulated DRG-cultures. Stimulation led to increased numbers of cells with higher fluorescent intensities. PMA activates Erk1/2 in virtually all neurons as nearly no cells remain with intensities of the control condition. D), E) Cell size profile of NGF/GDNF responding and non-responding cells. Responding neurons tend to be smaller in size. F), G) IB4 fluorescence intensity profile of NGF/GDNF responding and non-responding cells. NGF responding neurons tend to low intensity IB4 labeling while GDNF responding cells are mostly strongly IB4 labeled. H) Kinetic of NGF/GDNF induced Erk1/2 phosphorylation (n = 1000-4000 cells per time point, error bars SEM).

Above, we established the single neuron-based quantitative automated microscopy (QuAM) for unstimulated cultured DRG-neurons. Next we tested for detection of pErk1/2 intensities in response to known activators of Erk1/2, NGF and GDNF. In behavioral tests NGF- and GDNF-induced mechanical hyperalgesia was found to depend on activation of Erk1/2 [19,22]. Comparing the fluorescence intensities averaged over all neurons (n = 5203 cells for no stimulus, n = 3778 cells for NGF treatment, n = 1275 cells for GDNF treatment) we detected significant increases in Erk1/2 phosphorylation levels of about 6-fold after NGF treatment and of about 4-fold after GDNF treatment, respectively (Figure 2A).

Analysis of the data on a single cell base allowed deeper evaluation. The variability of the stimulated neurons was much larger than in the unstimulated cultures. Plotting the single neuron intensities of pErk1/2 in a scatter blot revealed increased intensities of up to 100-fold over average intensities of unstimulated cultures (Figure 2B). Nevertheless, a large number of cells showed intensities similar to the unstimulated neurons and thus were considered "non-responsive".

To test if all cells have the potential to be activatable we stimulated the culture with PMA. Phorbol esters such as PMA are very strong stimulants of Erk1/2 activation irrespective of upstream receptor expression. Therefore PMA stimulation should activate Erk1/2 in all cells. But PMA stimulation should allow also evaluation of the maximal responsiveness of sensory neurons as inactivation due to negative feedback loops should be circumvented. We found PMA stimulation of DRG-neurons to result in a complete shift of the pErk1/2 intensity histogram to higher intensities compared to the histogram of untreated cultures confirming that PMA is a strong Erk1/2 activating stimulus (Figure 2C).

To differentiate for each single neuron if it is activated or not, one can take the intersection between the intensity histogram of the unstimulated control cells and the stimulated cells as threshold value. While this will result in some false negatives this estimate is conservative in respect to the classification of truly positive i.e. activated neurons and thus allows deeper evaluation of parameters for activated neurons. Applying this method to PMA treated DRG-cultures we detected that single neuron intensities are for 92 ± 6% of the neurons higher than the intensities of unstimulated neurons (Figure 2C). This supports the previous assumption that PMA can activate Erk1/2 in all neurons.

The comparison of histograms of treated and untreated neurons allowed the definition of NGF and GDNF responsive neurons. We detected only 50 ± 8% NGF and 47 ± 8% GDNF responding cells, respectively, reflecting subgroup specific activation patterns (Figure 2c).

Next we compared the subgroups of NGF and GDNF responding cells in respect to their size. For NGF-responding cultures especially cells sized between 200 and 500 μm^2 ^were activated (Figure 2D). In contrast the GDNF responding cells had more frequently a size between 300 and 600 μm^2 ^(Figure 2E), suggesting that NGF and GDNF activate different cell populations.

### Subgroup specific activation of Erk1/2 can be defined through additional subgroup markers such as IB4

NGF and GDNF function has been shown to correlate with subgroup markers [16,19,38]. We employed the marker for GDNF-responsive neurons, the lectin IB4. The pErk1/2 and the IB4 fluorescence for each neuron were measured (Figure 2F, G). We analyzed the subgroups of NGF and GDNF responsive neurons in respect to the IB4 label intensity. The IB4 intensity histogram of NGF responding cells peaked at lower IB4 intensities indicating that most NGF responding cells are IB4 negative (Figure 2F). In contrast the IB4 intensity histogram of GDNF responding cells show less cells at lower but more at higher IB4 intensity level indicating IB4(+)-cells to be GDNF responsive (Figure 2G).

### NGF and GDNF induced pErk1/2 kinetics differ on a single cell base

Cells do not only differ in terms of baseline activity and responsiveness to stimuli. A very important parameter is also the pace of activation and signal transduction. As we now have established a technique to monitor the phosphorylation of Erk1/2, we therefore recorded next the kinetics of Erk1/2 activation on single neuron base. Beyond attempting a technical prove of principle this is also functionally of interest. As indicated in the introduction differential signalling kinetics of Erk1/2 can result in diametrically opposed cellular phenotypes induced by NGF versus EGF, respectively. Thus, for elucidating signalling mechanism it is not only important to investigate if but also when, how long, and how strong a kinase is activated [29,39].

We recorded for the first time the kinetics of endogenous Erk1/2 activation induced by the two sensitizing and receptor tyrosine kinase dependent growth factors, NGF and GDNF in primary DRG neurons. NGF showed a maximal Erk1/2 phosphorylation after 15 min maintaining a sustained high phosphorylation status thereafter (Figure 2H). Also GDNF resulted in phosphorylation of Erk1/2. Nevertheless, GDNF treatment resulted in a shallower kinetic curve representing a less strong and slower Erk1/2 activation than after NGF stimulation (Figure 2H).

### EGFR is expressed in DRG-neurons

We analyzed so far baseline activity of DRG-neuron subgroups and the activation kinetics in response to the sensitizing growth factors NGF and GDNF. This technique opens the door to elucidate the influence of other growth factors on Erk1/2, which are so far not described in the context of pain sensitization like EGF.

**Figure 3:**
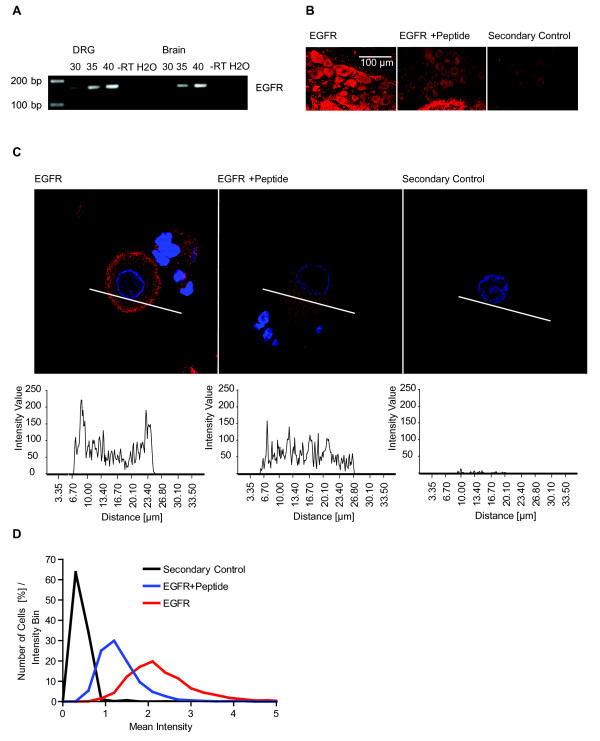
**EGFR is expressed in DRG-neurons**. A) Analysis of EGFR mRNA expression in lysates of DRG and brain from male rats by RT-PCR. RT-PCR shows a DNA-fragment with increasing intensity in dependence of the number (30, 35 and 40) of amplification cycles for DRG (lane 1-3) and brain (lane 6-8). Lane 4 and 9 reaction without reverse transcriptase (-RT) and lane 5 and 10 water control. B) Confocal images of DRG-sections. Left panel EGFR-staining, middle panel EGFR + blocking peptide, right panel secondary antibody control leaving out the primary antibody. C-terminal EGFR antibody blocking peptide was used in a 100× higher concentration than the antibody. C) Confocal images of DRG-cultures. Left panel EGFR-staining, middle panel EGFR + peptide, right panel secondary control. C-terminal EGFR antibody blocking peptide was used in a 100× higher concentration than the antibody. Intensity profiles along the indicated line crossing the cells indicate the plasma membrane staining of the EGFR-antibody. EGFR-C-terminal blocking peptide abolished the plasma membrane EGFR-signal D) QuAM quantified intensity histogram of EGFR-stained cultures (red), EGFR + blocking peptide (blue) and secondary control (black) indicated that EGFR is expressed in all neurons (n = 1874 cell for secondary control, n = 1720 cells EGFR, n = 1705 cells for EGFR + blocking peptide; p < 0.001).

First we tested, if EGF can potentially act directly on the nociceptive neuron by checking for expression of the EGF receptor (EGFR) in DRGs. RT-PCR showed an amplification of an EGFR specific fragment at the expected size indicating that EGFR is expressed on the level of mRNA (Figure 3A) in rat DRG (rat brain serving as positive control [40]).

But in DRGs there are also other cells than neurons such as glia and stellate cells. Thus, we analyzed the expression of EGFR in DRG-sections and dissociated DRG-neuron cultures using immunofluorescence microscopy (Figure 3B, C). DRG-sections as well as neuronal cultures showed higher fluorescence intensities and in case of cultures also a membrane located immunofluorescence signal if probed with an EGFR-specific antibody. EGFR-signals were detected in axonal fibers, glia-cells as well as in the DRG-neurons. To test antibody specificity we preincubated the EGFR-antibody with a blocking peptide. We observed a reduction of EGFR derived fluorescence intensities specifically in neuronal cell bodies and glia cells but not in the axonal fibers (Figure 3C). This identifies the cell body staining as specific while disregards the fiber staining as unspecific.

We also quantified the immunofluorescence intensities in DRG-cultures using QuAM (Figure 3D). The EGFR stained neurons showed significantly higher intensities than the cells stained only by the secondary antibody or by the EGFR antibody preincubated with a 100× higher concentration of the blocking peptide. There was a complete shift to higher intensities of the intensity histograms of EGFR stained cells compared to cells stained in presence of the blocking peptide. This indicated that in every single cell the EGFR-antibody derived intensity was higher than in the control cells. This suggests that EGFR is expressed not just in a subpopulation but in all DRG-neurons (Figure 3D).

### Phosphorylation of Erk1/2 differs strongly between NGF/GDNF and EGF treated neurons

We next investigated if we could detect an EGF induced increase in phosphorylation of Erk1/2. Because EGFR seemed to be expressed in all DRG-neurons (in contrast to the GDNF or NGF receptors described to be expressed only in a subset of cells [16,41]) we expected more EGF than NGF or GDNF responding cells. Therefore EGF stimulation should result in a higher number of cells showing an increasing pErk1/2 signal than in response to NGF or GDNF stimulation. Nevertheless, using 1 nM EGF we could not observe any pErk1/2 signal above baseline in contrast to stimulation with 1 nM NGF or 1 nM GDNF.

**Figure 4:**
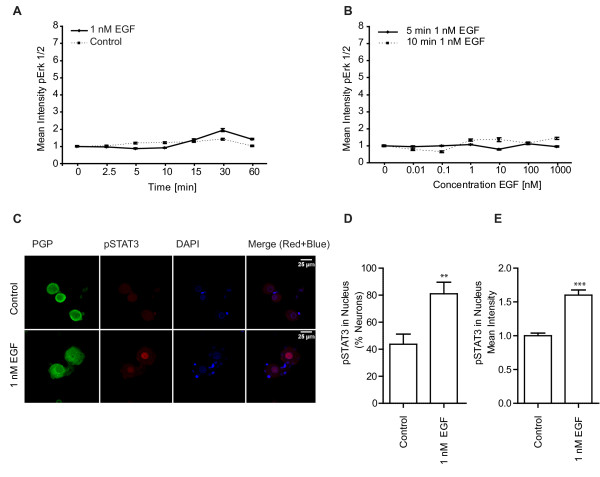
**EGF induces STAT3 translocation but not Erk1/2 phosphorylation**. A) Kinetic of phosphorylation of Erk1/2 in response to 1 nM EGF treatment of DRG-neurons (n = 1000-2000 cells for each time point, error bars SEM). B) Dose response curve after 5 min or 10 min stimulation (n = 1000-3000 cells for each concentration, error bars SEM). C) 1 nM EGF induced phosphorylation and translocation of STAT3 into the nucleus of DRG-neurons. D) Quantification of neurons showing phosphorylated STAT3 in the nucleus in response to 1 nM EGF (p < 0.01; n = 300 cells per condition were counted in 3 independent experiments). E) Mean intensity of pSTAT3 increased in nuclei of neurons in response to 1 nM EGF treatment (p < 0.01; Mean intensity of nuclei (61 (control) and 83 (EGF treated)) were quantified in 3 independent experiments).

We tried different time points of stimulation but with our current limit of sensitivity we could not detect any signal difference in comparison to the baseline signal (Figure 4A). 5 min EGF stimulation is reported to induce a maximal Erk1/2 phosphorylation in HeLa- cells [42] or PC12 cells [43]. Also stimulation times of 10 min are frequently used to detect Erk1/2 phosphorylation for example in astrocytes [44], fibroblast [45], kidney epithel cells [46] or Sertoli cells [47]. Thus, we tried different concentrations of EGF and stimulated for 5 min and 10 min (Figure 4B). But in contrast to NGF/GDNF, there is no EGF induced activation of Erk1/2 detectable in the heterogeneous system of DRG-neurons. Also starvation prior to EGF stimulation did not result in EGF induced Erk1/2 phosphorylation (data not shown).

To test if EGF initiates signalling in nociceptive neurons via a pathway different than the Erk1/2 pathway, we tested for the phosphorylation and translocation of the transcription factor STAT3. Indeed, stimulation of the nociceptive neurons with EGF (1 nM) for 15 min resulted in an increase of neurons showing pSTAT3 in the nucleus (Figure 4C). In addition, quantifying the intensity of pSTAT3 in EGF-treated versus untreated cells indicates a clear increase of the nuclear pSTAT3 signal (Figure 4D). Thus, apparently EGF does initiate signalling in DRG neurons but does not initiate phosphorylation of Erk1/2.

### EGF does not induce mechanical hyperalgesia

**Figure 5:**
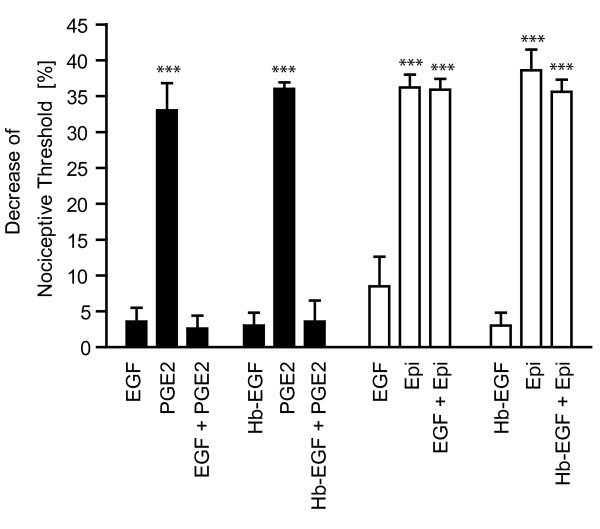
**EGF blocks PGE_2 _induced mechanical hyperalgesia**. Intradermal injection of 1 μg EGF and 1 μg Hb-EGF led not to a significant decrease in the nociceptive threshold in contrast to intradermal injection of 100 ng PGE_2 _or 100 ng Epinephrine. EGF and Hb-EGF abolished subsequent PGE_2 _but not epinephrine induced sensitization (p < 0.001). Reduction of paw-withdrawal threshold in EGF/PGE_2 _data series by 3.6 ± 1.9% after EGF treatment, 33.0 ± 3.8% after PGE_2 _treatment, 2.6 ± 1.3% after EGF+PGE_2_. (n = 6). Reduction of paw-withdrawal threshold in EGF/epinephrine data series by 8.5 ± 4.1% after EGF treatment, 36.2 ± 1.8% after epinephrine and 35.9 ± 1.5% after EGF+epinephrine (for all treatments n = 6). Reduction of paw-withdrawal threshold in Hb-EGF data series (3.0 ± 1.8% after Hb-EGF (n = 12), 36.0 ± 0.8% after PGE_2 _treatment, 3.6 ± 2.9% after Hb-EGF+PGE_2_, 38.6 ± 2.9% after epinephrine and 35.6 ± 1.7% after Hb-EGF+epinephrine (for all treatments n = 6)).

In our cell biological experiments we observed the cultured neurons to react differently to EGF than to NGF and GDNF stimulation, the former not activating Erk1/2 while the latter two leading to a sustained Erk1/2 phosphorylation. Therefore we hypothesized that EGF in contrast to NGF and GDNF will not lead to mechanical hyperalgesia. Intradermal injection of NGF and GDNF induces strong hyperalgesia as measured by the Randall-Selitto paw pressure test [19,22]. Indeed, as predicted from our cellular studies, intradermal injection of EGF or the physiological relevant EGFR agonist heparine bound EGF (Hb-EGF) led not to an establishment of mechanical hyperalgesia (no significant reduction of paw-withdrawal threshold by 8.5 ± 4.1% (or 3.6 ± 1.9% in other data series) after EGF treatment (n = 6) and 3.0 ± 1,8% after Hb-EGF (n = 12) (Figure 5).

### EGF inhibits PGE_2 _induced mechanical hyperalgesia

In PC12 cells NGF and EGF treatment evoked opposing responses, differentiation versus proliferation, respectively. Comparing EGF and NGF effects in pain sensitization we found that EGF in contrast to NGF treatment lead not to mechanical hyperalgesia. But absence of a response is unsatisfying. Accordingly, we tested if EGF treatment follows the example of PC12 cells and thus does the opposite of the NGF treatment. In the context of pain the opposite of "sensitization" is "block of sensitization". One classical inflammatory mediator resulting in sensitization is prostaglandin E2 (PGE_2_) [48]. Thus we tested, if pretreatment with EGF/Hb-EGF can block PGE_2 _induced mechanical hyperalgesia. Indeed EGF as well as Hb-EGF significantly abolished PGE_2 _mediated mechanical pain sensitization (reduction of paw-withdrawal threshold of EGF-data-series by 33.0 ±3.8% after PGE_2 _treatment, 2.6 ± 1.3% after EGF+PGE_2_, (for all treatments n = 6); reduction of paw-withdrawal threshold of Hb-EGF-data-series by 36.0 ± 0.8% after PGE_2 _treatment, 3.6 ± 2.9% after Hb-EGF+PGE_2_, (for all treatments n = 6)).

To investigate, if EGF shows general analgetic functions, we pretreated animals with EGF and Hb-EGF before injecting epinephrine. Epinephrine is an agonist of the β2-adrenergic receptor and described to induce mechanical hyperalgesia. In contrast to PGE_2 _induced mechanical hyperalgesia, EGF does not block the epinephrine mediated decrease of the nociceptive threshold (reduction of paw-withdrawal threshold of EGF-data-series by 36.2 ± 1.8% after epinephrine and 35.9 ± 1.5% after EGF+epinephrine; reduction of paw-withdrawal threshold of Hb-EGF-data-series by 38.6 ± 2.9% after epinephrine and 35.6 ± 1.7% after Hb-EGF+epinephrine (n = 6)).

## Discussion

### Investigation of heterogeneous DRG-neurons requires a defined quantitative microscopic approach

Standard methods to monitor the activation of signalling cascades like the Erk1/2 pathway are Western-Blot-analysis and ELISAs. Using these techniques cells are lysed and therefore information about single cells and subgroups is lost [49-51]. On the other hand, single cell based techniques such as FACS are not suitable for adherent cells. QuAM overcomes this problem. The establishment of such a quantitative microscopy approach provides two challenges: Neuron identification and quantification of fluorescence-derived signals. The automatic neuron recognition was challenged by the presence of glia-cells, fibroblasts and axon stumps in the DRG-culture. Nevertheless, the relative simple geometry of the cultured sphere-like neurons allowed the automatic detection of nearly all neuronal marker PGP 9.5 expressing neurons. Size and size distribution of the identified neurons resembles nicely published data [52].

Due to the automation, a large number of cells can be evaluated on a single cell base. Thereby, the distribution of the respective parameter can be exactly described. This allows evaluation, if the intrinsic assumption of the classical "eye-based" evaluation holds true, that a "marker-positive" and a "marker-negative" group can easily and clearly be separated as their respective intensity distributions are not or only slightly overlapping. Our first quantitative measurement of two such parameters, the phosphorylation status of Erk1/2 and the classical subgroup-marker, IB4 binding, shows clearly, that this assumption is not true. Thus, for any marker it is essential to define the distribution pattern and the respective threshold criterion to distinguish positive and negative cells. This result calls into question the validity of many of the published marker based subgroup quantifications.

Analysing the intensity distribution of the basal level of Erk1/2 phosphorylation, we find a very high degree of variability in the range of 180% and more. Nevertheless, despite the wide distribution we show that in principle subtle differences of a stable parameter such as size or marker-expression as well as changes of a fluctuating parameter such as activation state even as little as a 2% change (averaged over all cells) can be detected. Thus, the sensitivity of this quantitative approach is much increased over the current threshold based "by eye" analysis. In the latter case, one cannot evaluate for small shifts of distributions but rather is dependent on intensity increases in the range of multiples of the distribution deviation (e.g. 2 times 180%).

One problem central of heterogeneous systems is that one has to estimate the required sample size to reliably represent the whole divergent population. We find random sampling to nicely determine, how many cells have to be evaluated for which degree of sensitivity. Detection of changes such as a 22% of mean whole culture intensity requires a formidable number of 250 cells. Robust but subgroup specific changes require even more. For example, a 50% intensity increase in 1/5^th ^of the whole culture would result only in a whole culture intensity change of 10%. Thus, 1000 cells and more are required for detection.

To our knowledge our approach gives for the first time a rational to determine the required number of DRG-neurons to be evaluated. Our results also indicate the necessity to move forward to an automated quantitative high content analysis of this cellular system as manual counting of such large numbers of cells is in addition to being unreliable also extremely time consuming.

### Why to investigate endogenous signalling cascades

Most studies investigating e.g. growth factor induced signalling assume full responsiveness of all neurons expressing the respective growth factor receptor. But is this necessarily true? Recently, we showed responsiveness to ligands of the β2-adrenergic receptor to occur only in a subset of sensory neurons. Interestingly, while all neurons appear to express the receptor, only about 15-20% of the neurons responded by activation/translocation of the protein kinase C epsilon (PKCε) [31].

Interestingly, the number of responsive neurons could not be increased by circumventing the cascade initiating receptor by directly activating the downstream component Epac. Thus, while signalling cascades, of course, require the expression of a suitable receptor and its downstream components, this is not sufficient for ligand initiated signalling. We thereby gave proof of principle of a first IB4(+)-neuron specific signalling mechanism, which is not defined by receptor expression but by regulation of downstream signalling [31].

To what extent intracellular signalling depends on the cellular context only starts to emerge. One of the few other examples in nociceptors is the stunning effect of a NaV1.7 mutation. The very same point mutation resulted in a gain of function phenotype for nociceptive neurons but in a loss of function phenotype for sympathetic neurons [53]. Again, the cellular context defined in a drastic way the phenotypic result.

Detailed knowledge of context dependence for individual signalling pathways in single cells is so far rare in cell biology. Nevertheless, in non-nociceptive systems on a whole culture base there are some studies describing the signalling outcome to depend on the time, subcellular location and intensity of Erk1/2 activation [39]. A weak Erk1/2 signal in carcinoma cells led to apoptosis whereas a strong signal is related to cell survival [54]. Proliferation of fibroblast and differentiation of PC12 cells was proven to depend on localized signalling i.e. the translocation of Erk1/2 to the nucleus [55,56]. And in our eyes most stunningly, transient Erk1/2 activation results in proliferation, whereas sustained phosphorylation of Erk1/2 induced the opposite, namely differentiation of PC12 cells [29].

Thus, the description of signalling pathways and their kinetic properties does not merely fill a gap between stimulus input and effector output. To the contrary, it describes a complex computational mechanism which rules over effector activity in a non linear manner.

### Erk1/2 activation can define nociceptor subgroups

We have established with QuAM a technique with single cell resolution, which is able to correlate a number of cellular parameters with quantitative temporal and spatial resolution. To establish the ability to differentiate subgroup specific signalling we detected the phosphorylation level of the MAP kinase Erk1/2. Already in their basal state we find for the first time an indication, that IB4(+)-neurons differ in respect to their signalling status. The basal levels of Erk1/2 and more importantly for signalling also of phosphorylated Erk1/2 is much increased in IB4(+)- versus IB4(-)-neurons. As Erk1/2 has been shown to regulate a number of ion channels important in nociceptive signalling, this suggests a potential difference in basal ion channel properties. Indeed functional differences between IB4(+)- and IB4(-)-neurons have been reported. For example, the slow inactivation properties and the use-dependent inhibition of the voltage gated sodium channel NaV 1.8 are different depending on the expression of NaV 1.8 in either subgroup [57]. Some attempt to attribute the differences to differential ion channel expression [58]. But the activity of Erk1/2 has been associated tightly with the functionality of sensory neurons [9]. Thus, the altered basal activity status of Erk1/2 now detected by us, might indicate even beyond transcriptional/translational differences a novel reasoning for the observed differential functionality of IB4(+)-neurons, namely distinct signalling states.

We detected pErk1/2 levels also after treatment with the growth factors NGF, GDNF and EGF. The quantification of fluorescence intensities derived from phospho-specific antibodies in the obtained neurons showed indeed that stimulation with the growth factors NGF and GDNF lead to an increase in the phosphorylation signal of Erk1/2 in DRG-neurons but again only in subgroups of these sensory neurons. Our result of 50 ± 8% NGF-responding cells lay in the range of expression data for the NGF receptor TrkA, which is reported to be expressed primarily in peptidergic (IB4(-)) neurons [41]. The receptor for GDNF is a complex of a ligand binding domain GFR1-3 and the signal transducing transmembrane protein, RET, and is expressed in a subgroup of non-peptidergic (IB4(+)) neurons [16]. The subgroup size of IB4(+)-neurons differs in the literature from 40 - 70% most likely due to the semi-qualitative evaluation of a subjective experimenter [3-5]. Thus, our results of 71 ± 8% IB4(+)-cells and 47 ± 8% GDNF responding cells are in the range of published IB4-binding patterns and GDNF receptor expression data. In line with [2,3], which suggest that there is an overlap between IB4(+)- and CGRP/TrkA(+)-neurons, we observed a NGF response not exclusively in IB4(-)-neurons but also in IB4(+)-neurons. In contrast, the GDNF response was restricted with 90% nearly exclusively to IB4(+)-cells. After this proof of principle our quantitative approach now opens the door to describe new subpopulations on a novel base, namely on the base of signalling cascade activity.

### Growth factor signalling differs strongly in DRG-neurons

We could validate that our high content analysis and cell biology approach can help to elucidate roles for non investigated physiological relevant components such as the wound healing mediator EGF. It can help to formulate models for mechanisms of the sensitizing process and to explain behavioral phenotypes by gathering pathway activation data. The results were surprising. In contrast to the growth factors NGF and GDNF, EGF failed to induce Erk1/2 phosphorylation even though the EGFR is expressed in all DRG-neurons and the STAT3 signalling pathway is initiated by EGF in DRG-neurons. Commonly, receptor tyrosine kinase signalling is taken synonymous with Erk1/2 activation. But this might be a literature bias. Indeed, while there is strong pressure to report cellular responses to EGF in EGFR expressing cells, also other studies show EGFR expression [59,60] in adult DRG but fail to detect EGF induced Erk1/2 signalling activity in cell culture. Nevertheless, the EGFR agonists amphiregulin [61] and transforming growth factor [62] exert a clear survival effect. Along the same line, in developing P7-P9 DRG-neurons a slight increase in the phosphorylation signal of Erk1/2 after 5 min EGF stimulation could only be observed if using a tertiary antibody incubation step for additional signal amplification [63]. Not to forget, commonly even in clearly EGF responsive cell lines long-term starvation of the cells is prerequisite for signal detection (something not successful in nociceptive neurons (data not shown)). This indicates that while physiologically effective EGF induced signalling is not necessarily dependent on Erk1/2 activation. What is clear from our analysis is that the receptor tyrosine kinase induced signalling cascade toward Erk1/2 is activated fundamentally differently if initiated by EGF versus NGF and even GDNF. This indicates that growth factor function is growth factor specific in DRG-neurons.

### Role of EGF in pain can be predicted by QuAM

Our study proves, that the investigation of cellular signalling events can indicate a behavioral phenotype. As in the cellular experiments, EGF does not follow the growth factors NGF and GDNF and thus does not result in sensitization. In contrast and in correlation to observations in PC12 cells, EGF results in a phenotype opposing the one induced by NGF. Interestingly, EGF inhibits PGE_2 _but not epinephrine induced mechanical hyperalgesia. This argues against an unspecific effect of EGF such as general dampening of nociceptor activity. PGE_2 _is described to induce hyperalgesia by activation of PKA. In contrast epinephrine sensitizes only partly by activation of PKA but by activation of PKCε and Erk1/2 [34,48,64,65]. Which downstream signal of EGF induces this block has to be investigated in future. Nevertheless, our results are further proof that signalling cascades cannot be simply transferred from one cellular system to the next. Further signalling pathway outputs are highly stimulus dependent, even though it might involve similar components such as receptor tyrosine kinases like in the case of EGF, NGF and GDNF. QuAM elucidates these NGF/GDNF and EGF signalling differences and explains thereby the contrary behavior of these in inflammation physiological relevant growth factors.

With pain mechanisms one has to reflect about the potential use in humans. Are there indications for a similar action of EGF in humans? We believe so. If clinical studies of cancer patients treated with an EGFR inhibitor are analysed, one finds clear reports of therapy induced abdominal, chest and generalized pain [66-69]. This suggests that also in humans the presence of EGF is able to dampen nociception, as indicated by our study. Conversely, the block of EGF signalling apparently results in the patient in a removal of this break thereby inducing heightened sensitivity and pain.

## Conclusions

We established a microscopy based approach to detect the activation of endogenous signalling pathways on single cell level. Differences between stimulus responses could be analysed not only qualitatively but also quantitatively. This allowed us to define neuronal subgroups based on their signalling status in contrast to the common expression marker based subgrouping. Further, we found single cell derived activation status of signalling components to be an indicator for behavioral nociceptive phenotypes. If and how signalling cascades can be activated therefore adds a novel layer of complexity to nociceptor functionality, which so far is mostly defined just by ion channel expression. In addition, we demonstrated that EGF show different functionality than NGF or GDNF and that EGF is a new potential analgesic modulator in the process of pain sensitization, a result of importance not least in the light of the increased use EGFR blockers for therapeutic cancer treatment.

## List of abbreviations used

DRG: dorsal root ganglia; EGF: epidermal growth factor; EGFR: epidermal growth factor receptor; Epi: Epinephrine; f.c. final concentration; GDNF: glial cell line-derived neurotrophic factor; Hb-EGF: heparin bound epidermal growth factor; MAPK: mitogen activated protein kinase; NGF: nerve growth factor; IB4: isolectin B4; PBS: Phosphate buffered saline; PC12: cells pheochromocytoma cells; pErk1/2 (Thr-202/Tyr-204) phosphorylated Erk1/2; PGE_2_: prostaglandin E2; PKC: protein kinase C; PMA: phorbol 12-myristate 13-acetate; QuAM: quantitative automated microscopy; RT: Room temperature.

## Competing interests

The authors declare that they have no competing interests.

## Authors' contributions

CA designed and performed the experiments and wrote the manuscript. SM designed and performed the data analysis by random sampling. OD and JD designed and performed the behaviour experiments. TH initiated the studies, designed the experiments and wrote the manuscript. All authors have read and approved the final manuscript.

## Supplementary Material

Additional file 1 **Supplementary figure 1, Automatic control of exposure time does not influence normalized signal intensity**. The DRG-culture was imaged with 0.96s six times until the fluorescence signal was not decreasing anymore. Then the culture was imaged with different usually used exposure times (between 0.24s and 0.96s) in a different sequence. The mean intensities + standard deviations of 10 single cells, identified by their position on the slide, were compared to observe any exposure time dependent intensity differences. There was no significant difference of signal intensities in dependence of different exposure times. Intensity values: 0.96 s: 0.384 ± 0.118; 0.48 s: 0.386 ± 0.118; 0.24 s: 0.396 ± 0.120; 0.48 s: 0.385 ± 0.120; 0.96 s: 0.378 ± 0.118; 0.24 s: 0.391 ± 0.117. Click here for file
