# Supplementary material for: Quantitative automated microscopy (QuAM) elucidates growth factor specific signalling in pain sensitization
Source: Mol Pain. 2010 Dec 27;6:98. doi: 10.1186/1744-8069-6-98 (PMC3023724; doi:10.1186/1744-8069-6-98)
Supplement: Additional file 1 — Supplementary figure 1, Automatic control of exposure time does not influence normalized signal intensity. The DRG-culture was imaged with 0.96s six times until the fluorescence signal was not decreasing anymore. Then the culture was imaged with different usually used exposure times (between 0.24s and 0.96s) in a different sequence. The mean intensities + standard deviations of 10 single cells, identified by their position on the slide, were compared to observe any exposure time dependent intensity differences. There was no significant difference of signal intensities in dependence of different exposure times. Intensity values: 0.96 s: 0.384 ± 0.118; 0.48 s: 0.386 ± 0.118; 0.24 s: 0.396 ± 0.120; 0.48 s: 0.385 ± 0.120; 0.96 s: 0.378 ± 0.118; 0.24 s: 0.391 ± 0.117. [file 1744-8069-6-98-S1.PDF]

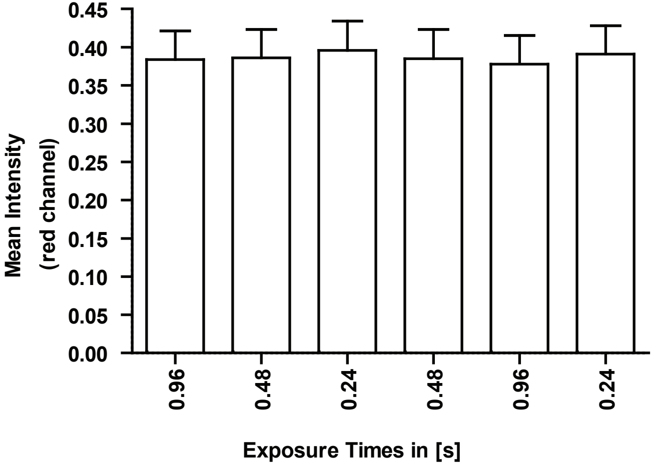

The DRG-culture was imaged with 0.96 s six times until the fluorescence signal was not decreasing anymore. Then the culture was imaged with different usually used exposure times (between 0.24 s and 0.96 s) in a different sequence. The mean intensities + standard deviations of 10 single cells, identified by their position on the slide, were compared to observe any exposure time dependent intensity differences. There was no significant difference of signal intensities in dependence of different exposure times. Intensity values: 0.96 s: 0.384 ± 0.118; 0.48 s: 0.386 ± 0.118; 0.24 s: 0.396 ± 0.120; 0.48 s: 0.385 ± 0.120; 0.96 s: 0.378 ± 0.118; 0.24 s: 0.391 ± 0.117;
